# The Initial Oral Microbiota of Neonates Among Subjects With Gestational Diabetes Mellitus

**DOI:** 10.3389/fped.2019.00513

**Published:** 2019-12-10

**Authors:** Zhijiang He, Jiaman Wu, Bin Xiao, Shanqiu Xiao, Hongping Li, Kaifeng Wu

**Affiliations:** ^1^Shenzhen University General Hospital, Shenzhen, China; ^2^Affiliated Shenzhen Maternity and Child Healthcare Hospital, Southern Medical University, Shenzhen, China; ^3^Shenzhen Children's Hospital, Shenzhen, China; ^4^Bao an Maternal and Child Health Hospital, Jinan University, Shenzhen, China; ^5^Boluo Maternal and Child Health Family Planning Service Centre, Huizhou, China

**Keywords:** gestational diabetes mellitus, neonatal oral microbiome, 16S rRNA sequencing, oral microbiota, neonate

## Abstract

**Objective:** The objective was to investigate the potential effect of gestational diabetes mellitus on the initial neonatal oral microbiome community structure.

**Methods:** Oral samples were collected from 20 full-term, vaginally delivered newborns with sterile swabs. Nine of them had mothers diagnosed with gestational diabetes mellitus (GDM group), while 11 had non-diabetic mothers (NDM group). The oral microbiota was analyzed using multi-barcode 16S rRNA sequencing on Illumina MiSeq system.

**Results:** The results showed that the birth weight, gestational age and gestational weight gain were significantly higher in NDM group. There was a significant correlation between gestational age and birth weight. Neonatal oral microbiome was composed of five dominant phyla from *Actinobacteria, Bacteroidetes, Firmicutes, Proteobacteria*, and *Tenericutes*. Compared to NDM group, a higher alpha diversity and reduction of phylum *Firmicutes* were observed in GDM group. Genus *Lactobacillus* dominated in NDM group, while *Alistipes, Streptococcus*, and *Faecalibacterium* were overabundant in GDM group. Additionally, carbohydrate metabolism increased in NDM group, whereas amino acid metabolism, vitamin metabolism and lipopolysaccharide biosynthesis were more abundant in GDM group.

**Conclusions:** This study showed a distinct oral microbiota profile in neonates born to mothers with GDM, which indicated that maternal diabetes status played an important role in neonatal initial oral microbiota.

## Introduction

Gestational diabetes mellitus (GDM) is a high-risk obstetric complication that characterized by glucose intolerance during pregnancy (1). Along with increasing rates of obesity, its incidence increases dramatically (2). The International Diabetes Federation estimated in 2017 that one seventh of live births worldwide were affected by GDM (3). Infants born to mothers with GDM had increasingly high risk of developing preterm birth, shoulder dystocia, caesarian section, and metabolic dysfunction such as neonatal hypoglycemia (4, 5). In addition, long-term health risks were observed from mothers, including the high probability on developing obesity, insulin resistance, and type 2 diabetes mellitus (5, 6). Therefore, GDM not only increased adverse pregnancy outcomes of newborns, but also had long-term deleterious effects on maternal metabolic health.

Accumulating evidence demonstrated that disruption of human microbiota across multiple sites such as gut, skin, mouth, and so on, were linked to lots of diseases and symptoms (7). And the early time of life was the critical period for colonization and maturation of intestinal microbiota, which could not only affecting the maturation of neonatal immune system, but also influencing subsequent development of diabetes (8, 9). The disturbances of intestinal microbiota might disrupt the normal function of immune system and metabolic status (10). Previous study showed that diversified structures and function of intestinal microbiome community existed in newborns born to GDM mothers (11). And the placental microbiota also altered among subjects with GDM (12). As an important organ for fetal development, there is lack of understanding on oral microbiome in newborns with GDM mothers.

Oral microbes contributed strongly to shape human health, its dysbiosis in early life related to many oral diseases, including dental caries, periodontitis, and oral mucosal diseases (13, 14). Moreover, dynamic changes were observed in oral bacterial composition during pregnancy and childhood development (15). In particular, the first microbial colonizers of the oral cavity in the first day of life had an important impact on the growth of subsequent species (16). In this light, there were growing interests in understanding which endogenous and environmental factors influence the composition of the neonatal oral microbiome. More and more studies demonstrated that numerous factors were related to the initial colonization of the first oral microbiome (17, 18), especially the status of mother, which was the primary source (19).

GDM develops during pregnancy present a particularly alarming trend for its increasing rate (12). And as a high risk factor of neonatal and maternal health, the relationship between GDM and neonatal oral microbiome is poorly understood. Therefore, the main objective of this study is to characterize the composition of the neonatal oral microbiota and further to assess whether maternal diabetes status affects its composition.

## Methods

### Subjects and Samples

The prospective randomized pilot study enrolled 20 full-term and vaginal delivery newborns, and it was conducted at Bao an Maternal and Child Health Hospital (Shenzhen, China) in 2016. For enrollment criteria, (1) infants born with gestational age from 37 to 42 weeks, (2) birth weight >2,500 g, (3) without any significant congenital anomalies, neurological dysfunction, fetal chromosomal abnormalities, metabolic diseases or need resuscitation after birth. The exclusion criteria were mothers without these clinical conditions: chorioamnionitis, preeclampsia, eclampsia, maternal hypertension, maternal obesity, infections, autoimmune diseases, uterine malformation, cancer, or any other systemic diseases. Maternal and neonatal clinical characteristics include age, pre-pregnancy BMI, antepartum BMI, gestational weight gain, birth weight and gestational age, were collected from standardized medical records. The ethical committee of hospital approved all protocols. Signed informed consents were obtained from parents or legal guardians of all participants.

GDM was diagnosed with the fasting plasma glucose ≥5.1 mmol/L or 1 h post-OGTT glycaemia ≥ 10 mmol/L or 2 h post-OGTT glycaemia ≥8.5 mmol/L, according to the criteria set by International Association of Diabetes and Pregnancy Study Groups (20). The control participants had a normal 2-h 75 g oral glucose tolerance test. To ensure the stability of glucose level, only exercise and diet control, without insulin were used to treat GDM participants. They received dietary counseling and nutritional recommendations in line with clinical guidelines. And 30-min daily moderate exercise was recommended.

All samples were collected by trained nurses according to the previously described protocol (18). During the sample collection process, nurses wore facial masks and sterile gloves and mothers did not handle to avoid possible contaminations. The neonatal oral samples were collected with sterile swabs 1 min after birth. The swabs were placed in cell lysis solution (1,000 μL) immediately after collecting and then stored in −80°C freezer for DNA extraction.

### Sequencing and Sequence Processing

DNA was isolated from oral samples using commercially available kit (Qiagen) according to manufacturer's instructions. After extracted DNA purified, its concentration and quality were determined by Qubit (Invitrogen) and verified with agarose gel electrophoresis. 16s rRNA V3-V4 variable region was amplified using forward primer 5′CCTACGGGNGGCWGCAG3′ and 5′ reverse primer. Replicate amplicons for each sample were equally pooled for sequencing. Paired-end sequenced on an Illumina MiSeq instrument with barcoding using sequence kit version 3.0 to achieve the desired reads.

FastQC (http://www.bioinformatics.babraham.ac.uk/projects/fastqc/) was initially used to evaluate the sequence data quality. De-multiplexing was then performed based on custom Perl scripts with 1 mismatch allowing. Mothur pipeline was used to handle and analysis the high quality sequencing data (21). Firstly, paired-end reads were merged into tags, removing tags either with high amount of ambiguous bases and homo-polymers, or out of expected range. Secondly, tags were aligned to SILVA 119 database (22), only kept with correct alignment region and coordinates. Followed by de-replicating and de-noising processes. Next, the chimeric sequences were discarded based on UCHIME with reference database mode (23). Then substantial taxonomic classifications were performed using Ribosomal Database Project (RDP) Naive Bayesian Classifier with an 80% pseudo-bootstrap confidence score (24). After removing sequences without bacterial assignment, the rest sequences were grouped to operational taxonomic units (OTUs) at 97% similarity level.

### Statistical Analysis

Continuous characteristics were reported as means ± standard deviation (SD), whilst categorical variables were presented as ratios. The statistical analysis was performed using R software. Unpaired *t*-tests and Fisher's exact tests were used to study differences in continuous and categorical data separately between GDM and NDM groups. Furthermore, the relationships between microbial taxa and gestational age were identified by Pearson correlation coefficient. *P* < 0.05 was considered to be statistically significant.

The LEfSe (linear discriminant analysis effect size) algorithm (25) was used to determine the differences in bacterial composition at various taxonomy levels between GDM and NDM groups.

Extended error bar plot was generated using the STAMP program (26), which showed significantly different features between GDM and NDM groups with *P* < 0.05.

## Results

### Characteristics of the Infants

Clinical characteristics of mothers and infants were shown in Table 1, which were obtained by a trained nurse. All infants were from Chinese Han ethnicity, vaginally delivered at full-term, with gestational age between 37 and 42 weeks. The average value of gestational age was 38.94 ± 2.75 weeks, that of birth weight was 3121.55 ± 324.67 g, and with 45% boys. Those counterparts were compared between GDM and NDM groups. The gestational age, birth weight, and gestational weight gain significantly increased in NDM group. No significant differences were observed in maternal age, pre-pregnancy BMI, antepartum BMI, fasting glucose level, and ratio of boy between infants with and without diabetic mothers (all *P* > 0.05). Average maternal age was 29 for participants in both groups.

**Table 1:** Characteristics compared between the GDM and NDM group.

	**GDM group (*N* = 9)**	**NDM group (*N* = 11)**	***P*-value**
**Newborn's characteristics**			
Gestational age (week)	38.81 ± 1.21	39.96 ± 0.86	0.02
Birth weight (g)	2955.67 ± 296.56	3257.27 ± 291.38	0.03
Male/female	3/6	6/5	0.62
**Mothers' conditions**			
Pre-pregnancy BMI (kg/m^2^)	21.22 ± 5.59	19.05 ± 2.22	0.30
Antepartum BMI (kg/m^2^)	23.74 ± 1.95	25.17 ± 3.30	0.61
Gestational weight gain (kg)	10.5 ± 3.35	14.89 ± 4.48	0.02
Fasting glucose (mg/dL)	6.74 ± 2.07	4.70 ± 0.72	0.13
Age (year)	28.44 ± 3.43	28.64 ± 3.17	0.90

*Fisher's exact test was used for categorical variables and t-test was used for continuous variables. P < 0.05 was considered as significant*.

### GDM and NDM Infants Showed Significant Difference on Oral Microbial Diversity

The oral samples were collected from 9 infants born to mothers with gestational diabetes mellitus and 11 infants born to non-diabetic mothers. Amplified by the universal bacterial 16s rRNA primers, the positive PCR products of V3-V4 region were then sequenced on the Illumina MiSeq platform. Totally, 1,239,170 sequences with an average of 61,958 sequences per sample were gained.

**Figure 1:**
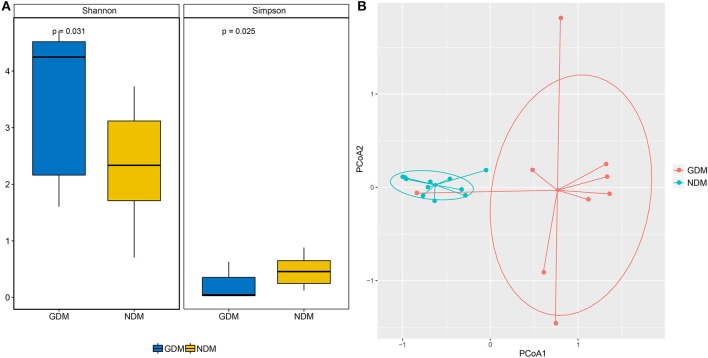
Comparison of the microbiome biodiversity between GDM and NDM groups. **(A)** The Shannon index, Simpson index were shown as estimators to demonstrate the difference of alpha diversity between the two groups. **(B)** Principal coordinate analysis plot based on the abundance and diverse in neonatal oral microbiomes between the two groups. Each point represents the oral microbiota of a newborn.

The Shannon and Simpson indices were applied to evaluate microbial diversity. Based on the OTUs distribution, the average value of Shannon index was 3.48 ± 1.29 (mean ± SD) and 2.30 ± 0.97 in GDM and NDM groups, respectively (*P* < 0.05). The Simpson index also reflected significant discrepancy: averaging 0.20 ± 0.24 for infants born to mothers with gestational diabetes mellitus and 0.47 ± 0.25 for infants born to mothers with normal glucose (*P* < 0.05) (Figure 1A). Both Shannon and Simpson indices showed significantly statistical difference between GDM and NDM groups, and GDM group had higher alpha-diversity than NDM group.

To compare overall initial oral microbiota structure in neonates born to mothers with and without gestational diabetes mellitus, principal coordinates analysis (PCoA) according to the relative taxa abundance was implemented. The results of PCoA analysis exhibited a significant separation in bacterial composition between GDM and NDM groups (Figure 1B). Most of the subjects in different group were well-separated, only GDM4 showed no separation with subjects of NDM group. And the subjects of GDM group were quite distant from each other based on the thetayc dissimilarity. It indicated that there was a significant clustering based on maternal diabetes status.

### Overall Microbial Structures of Oral Microbiome

**Figure 2:**
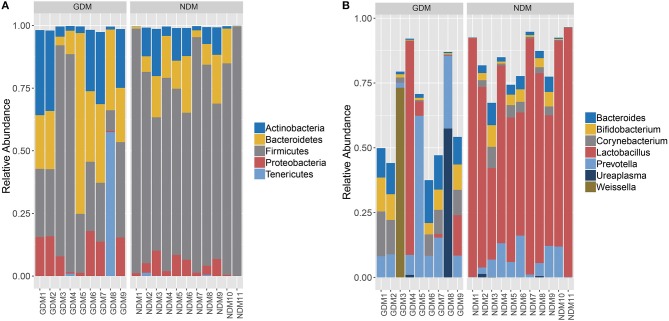
Overal structures of oral microbiomes of each enrolled subjects. **(A)** Relative abundance at the phylum level. **(B)** Relative abundance at the genus level.

The overall microbiota compositions of each sample at the phylum and genus levels were shown in Figure 2. The main phyla of both two groups were *Actinobacteria, Bacteroidetes, Firmicutes, Proteobacteria*, and *Tenericutes*, which accounted for about 99% of the microbial content (Figure 2A). Phylum *Firmicutes* was the most abundant in all enrolled subjects and *Tenericutes* rarely presented. Compared to NDM group, there was an approximately 38.74% decrease in the average proportion of *Firmicutes* (38.48 vs. 77.22%) in GDM group, whereas the mean proportion of *Actinobacteria* (16.94 vs. 7.15%), *Bacteroidetes* (27.0 vs. 10.76%), *Proteobacteria* (9.87 vs. 4.04%), and *Tenericutes* (6.50 vs. 0.19%) increased with almost the same percentage.

At the genus level (Figure 2B), seven major genera, *Bacteroides, Bifidobacterium, Corynebacterium, Lactobacillus, Prevotella, Ureaplasma*, and *Weissella* were represented. Specifically, the proportion of *Lactobacillus* accounted for more than 69.06% in NDM group, higher than that in GDM group (11.76%). Compared to NDM group, the mean proportion of *Prevotella* (16.60 vs. 6.80%)*, Bacteroides* (7.46 vs. 2.98%)*, Bifidobacterium* (5.31 vs. 2.81%)*, Corynebacterium* (6.68 vs. 2.45%)*, Ureaplasma* (6.49 vs. 0.19%), and *Weissella* (8.14 vs. 0.01%) increased in GDM group.

### Correlation Between Oral Microbiota and Gestational Age

**Figure 3:**
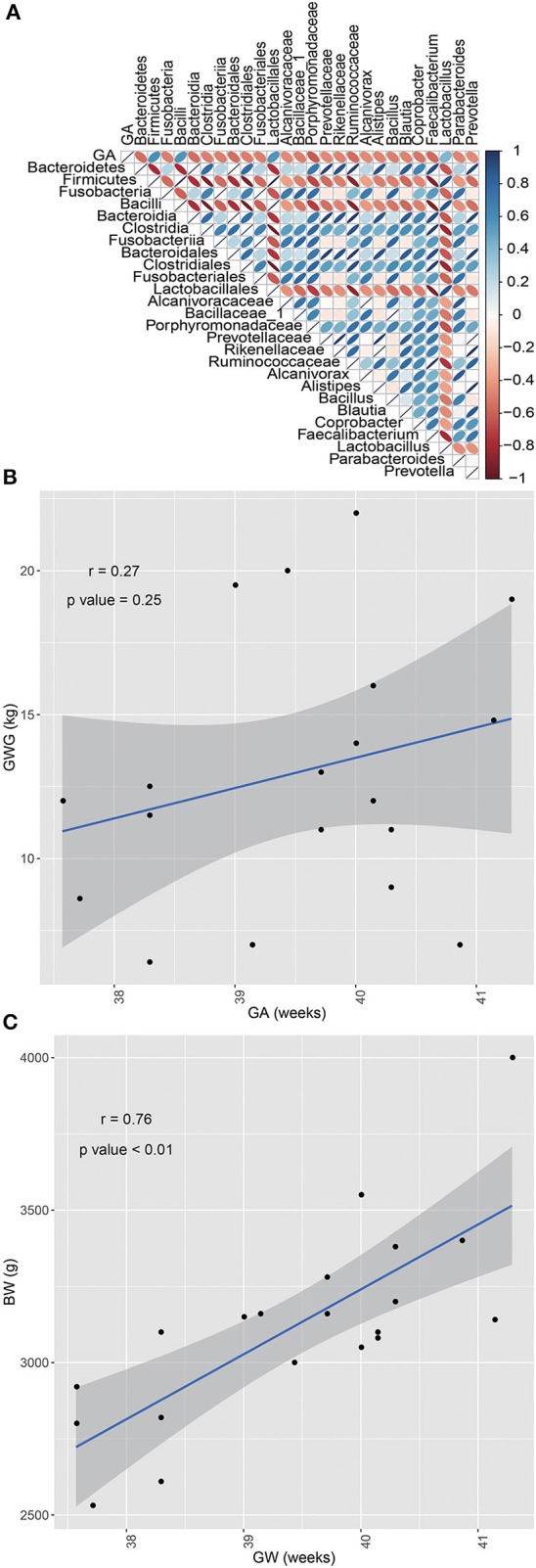
Results of correlation analyses. **(A)** Correlation analyses between oral microbiota and gestational age. Taxonomy significantly correlated with gestational age. **(B)** Correlation analysis between GWG and GA, without significant correlation. **(C)** Correlation analysis between BW and GA, that presented a significant correlation.

As the characteristic analysis showed that significant higher gestational age was observed in NDM group than GDM group. Next, specific oral microbiota that potentially correlated with gestational age was investigated. Pearson's correlation analysis was performed between all taxonomy and gestational age. The absolute value of *r* > 0.5 and *P* < 0.05 were used as filter parameters. The results showed that phyla *Bacteroidetes, Firmicutes*, and *Fusobacteria* were significantly associated with gestational age. Additionally, genera *Alcanivorax, Alistipes, Bacillus, Blautia, Coprobacter, Faecalibacterium, Lactobacillus, Parabacteroides*, and *Prevotella* were correlated with gestational age significantly (Figure 3A).

Table 1 showed that gestational age (GA, weeks), birth weight (BW, g), and gestational weight gain (GWG, kg) were significantly different between GDM and NDM groups. In order to figure out whether GWG and BW associated with GA, respectively, the Person correlation analysis between GWG and GA, BW and GA were performed. Figure 3C indicated that BW significantly correlated with GA, whereas no statistically significant correlation was observed between GWG and GA (Figure 3B).

### Taxonomic Comparisons of Oral Microbiota Between GDM and NDM Groups

**Figure 4:**
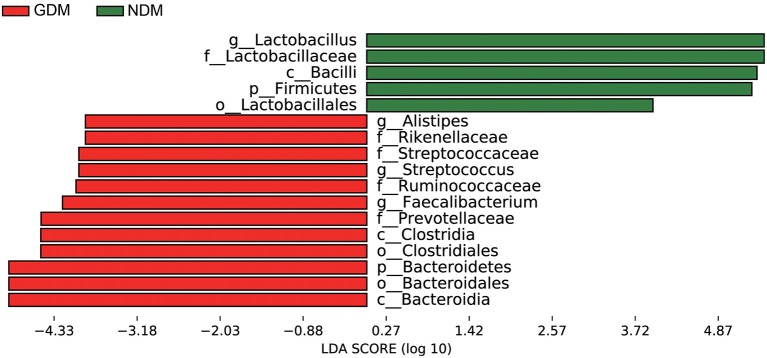
LEfSe analysis of oral microbiota in neonates born to mothers with and without gestational diabetes mellitus. Histogram of the LDA scores generated by LEfSe indicating differences at phylum, class, order, family, and genus levels between the two groups, the prefixes “p,” “c,” “o,” “f,” “g” represent the annotated level of phylum, class, order, family and genus.

LEfSe analysis suggested that significant differences were observed in the taxonomic composition of oral microbiota from the phylum level down to the genus level between infants born to mothers with and without gestational diabetes mellitus (Figure 4). It depicted all bacteria that showed significant difference (*P* < 0.05) between GDM and non-diabetic controls. At the phylum level, *Bacteroidetes* was significantly enriched in GDM group, whereas the relative abundance of *Firmicutes* was higher in NDM group. At the class level, enrichments of *Bacteroidia, Clostridia* in GDM group and *Bacilli* in NDM group were observed. And at the genus level, *Alistpes, Streptococcus* and *Faecalibacterium* exhibited relatively higher abundance in GDM group, while *Lactobacillus* was relatively more abundant in NDM group.

### Functions of Metabolism in the Oral Microbiome of GDM and NDM Neonates

**Figure 5:**
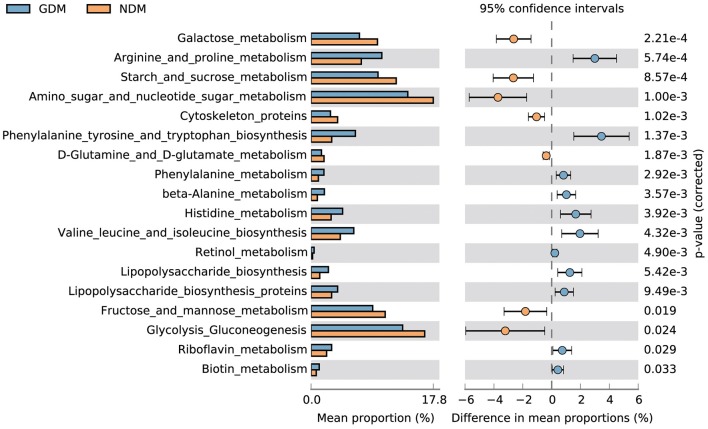
Functional properties of neonatal oral microbiota that differ significantly between GDM and NDM groups.

To understand the GDM induced metabolic alterations in neonatal oral microbiome, functional profiles from 16S sequencing data were derived with PICRUST program (27) based on the Kyoto encyclopedia of genes and genomes (KEGG) database. Next, differences of KEGG pathways between the two groups were analyzed with STAMP (26). The results demonstrated that most differences in vitamin, amino acid and carbohydrate metabolism pathways, which showed in an extended error bar (Figure 5). Compared to NDM group, vitamin metabolism, amino acid biosynthesis and amino acid metabolism in GDM group increased significantly. On the other side, carbohydrate metabolism was more abundant in infants with non-diabtetic mothers. In addition, significant down-regulation of lipopolysaccharide biosynthesis, lipopolysaccharide biosynthesis proteins were observed in NDM group, and phosphotransferase system was significantly down regulated in GDM group. Furthermore, some molecules related to membrane and intracellular structure, were significantly different between GDM and NDM groups, such as glycosphingolipid biosynthesis and cytoskeleton proteins and so on.

## Discussion

GDM has a long-term impact on the health of mother and children. To investigate the effect of GDM on neonatal initial oral microbiome, 16S rRNA sequencing was implemented using the Illumina MiSeq system to explore the initial oral microbiota profile of neonates born to mothers with gestational diabetes mellitus and it's discrepancy to neonates born to non-diabetic mothers. Several studies indicated that the mode of delivery and low birth weight could affect the gut and placental microbiome of neonates (28, 29), which might affect neonatal initial oral microbiota. Thus, only neonates with vaginal delivery and birth weight >2,500 g were recruited to minimize the impact of potential factors in this study.

In this study, a total 1,239,170 high-quality sequences were produced and 61,958 reads per sample were analyzed, suggesting that was useful in estimation of the details of oral bacterial microbiota at a more in-depth level. In accordance with previous study (30), the taxonomy analysis demonstrated that the oral microbiota was composed of five dominant phyla from *Firmicutes* (the most abundant), *Actinobacteria, Bacteroidetes, Proteobacteria*, and *Tenericutes*, with the proportion of *Firmicutes* decreased and the others phyla increased in GDM group compared to those of NDM group. Since *Actinobacteria, Bacteroidetes, Firmicutes*, and *Proteobacteria* also dominated in women's placentas, the proportion of *Firmicutes* decreased and *Proteobacteria* increased in women with GDM (12). Another previous study also reported that 42 women with GDM had a low relative abundance of *Firmicutes* in gut (30). Moreover, *Firmicutes* had also been seen in low abundance in cohorts of type 2 diabetes patients (9, 31). To sum up, the similar trend of microbial varying across the maternal and neonatal microbiota were observed. And Aagaard et al. reported placental microbiome profile was most akin to the oral microbiome profile (32). There might be some transmission routines from maternal placental and gut microbiota to neonatal oral cavity. However, no causal relationship could be inferred from this study, and the increase of *Proteobacteria* in GDM group might sign the dysbiosis of oral microbiota (33). Thus, more evidences and further investigations were needed to study the causal relationship, and to understand the effect of initial oral bacterial dysbiosis on neonatal health.

A significantly higher alpha-diversity of infants born to mothers with gestational diabetes was observed compared to infants born to non-diabetic mothers. And PCoA analysis demonstrated that subjects of GDM group separated from those of NDM group. Further analysis showed that significant variations in the composition of oral microbiota were observed between the GDM and NDM group from the phylum level down to the genus level. Increasing of phylum *Bacteroidetes* and decreasing of phylum *Firmicutes* were concordant with a previous study in GDM group (31). At the genus level, the dominant genus in NDM group was *Lactobacillus*, which was consistent with the recent finding that suggested certain species of *Lactobacillus* were more prevalent in neonatal oral microbiota (34). As members of probiotic, *Lactobacillus* has positive effect on the host health, because it produces many beneficial organic acid lactates and these can be converted into butyrate (35). High abundance of genus *Alistipes* was seen in some autism children (36), and it was associated with abdominal pain in children (37). In addition, genus *Faecalibacterium* had been reported prevalent in women with gestational diabetes (38), and genus *Streptococcus* were more abundant in adult diabetes cases (39). In this study, the proportion of *Lactobacillus* decreased, whereas *Alistipes, Faecalibacterium*, and *Streptococcus* increased dramatically in GDM group. These variations might be related to diabetes and other disorders, which would pose GDM newborns at a higher risk of developing these diseases in the future than control newborns.

Previously, it was reported that genus *Alistipes* and *Lactobacillus* in maternal gut were both correlated with GWG (38). And a positive relationship existed between *Alistipes* in placental microbiota and GWG (12). However, the results in this study showed that *Alistipes* (*r* = −0.31, *P* = 0.18) and *Lactobacillus* (*r* = 0.44, *P* = 0.05) in neonatal oral cavity were not significantly associated with GWG. Santos et al. noted that a small part of infants with large gestational age were attribute to excessive GWG (40). Additionally, gestational age also played a key role in birth weight (41). Then the relationships among them were investigated in this study. Non-significant association between GA and GWG, significant correlation between GA and BW were observed. On the other side, 3 phylum and 9 genera including *Alistipes* and *Lactobacillus* were significantly correlated with GA. This demonstrated more than gestational age, multiple factors such as microbiota and so on, impacted gestational weight gain during pregnancy (42). Furthermore, the results in this study showed that birth weight of neonates, gestational age and gestational weight gain of mothers were significantly higher in the NDM group, compared to the GDM group. Collectively, except neonatal development, GDM also affected the initial bacterial colonizer of neonatal oral microbes. Limited by the lack of follow-up data, we had no idea about its effect on subsequent stage of newborn lives. Thus, a large cohort of samples and longitudinal data should be collected to confirm these findings, and to figure out how GDM impact the health of newborns.

Women with GDM had higher level of asparagine, tyrosine, valine, phenylalanine, glutamic acid, and isoleucine (43). In concordance with previous study, oral microbial genes in GDM group enriched in amino acid, vitamin, and lipopolysaccharide biosynthesis in this study, while not active in carbohydrate metabolism enzymes, such as galactose metabolism and starch and sucrose metabolism. Enquobahrie et al. demonstrated that amino acids metabolism deregulation was related to insulin resistance and related disorders, and a high part of d-galactose in women with GDM was observed (44). The deficiency of carbohydrate metabolism in woman with GDM might impact their postprandial glycemic response. Additionally, lipopolysaccharide is a major component of the outer membrane of gram-negative bacteria, and it plays an important role in the pathogenesis of certain bacterial infections as endotoxin (45). Its enrichement in GDM group might have important effects on neonatal health, and its long-term clinical consequences need to be further followed.

However, the limitations of this study should be taken into consideration. Limited sample number and all participants from the same hospital might affect the results. Our ongoing study is aimed at enlarging the sample size. Limited sampling time hindered the understanding of long-lasting effects on oral microbiota development of infants born to mothers with GDM. And this cross-section study was not enough to infer the causality between GDM and neonatal oral microbiome. Moreover, after birth, a lot of factors would impact the neonatal oral microbiota, such feeding type, mother status, environment and so on. Therefore, a multicenter clinical study among different regions and well-controlled longitudinal studies should be design. And in order to clarify the path of mother-to-baby efflux of commensal microbes during pregnancy, prospective studies would be required.

In summary, this study demonstrated that maternal gestational diabetes was associated with an aberrant oral microbial composition of newborns. Furthermore, the oral microbiome of infants born to mothers with GDM was enriched for the bacteria observed in the gut microbiome of gestational diabetes patients. These findings in this study could enhance our understanding of the colonization of neonatal oral microbome.

## Data Availability Statement

The raw data supporting the conclusions of this manuscript will be made available by the authors, without undue reservation, to any qualified researcher.

## Ethics Statement

The studies involving human participants were reviewed and approved by Ethical Committee of Bao an Maternal and Child Health Hospital, Jinan University (Shenzhen, China). Written informed consent to participate in this study was provided by the participants' legal guardian/next of kin.

## Author Contributions

KW and HL conceived of the presented idea and planned the experiments. ZH, JW, and SX carried out the experiments. HL and BX designed the computational framework and analyzed the data. ZH and JW wrote the manuscript. All authors discussed the results and contributed to the final manuscript.

### Conflict of Interest

The authors declare that the research was conducted in the absence of any commercial or financial relationships that could be construed as a potential conflict of interest.
